# Social mixing patterns in rural and urban areas of southern China

**DOI:** 10.1098/rspb.2014.0268

**Published:** 2014-06-22

**Authors:** Jonathan M. Read, Justin Lessler, Steven Riley, Shuying Wang, Li Jiu Tan, Kin On Kwok, Yi Guan, Chao Qiang Jiang, Derek A. T. Cummings

**Affiliations:** 1 Department of Epidemiology and Public Health, Institute of Infection and Global Health, University of Liverpool, Leahurst Campus, Neston CH64 7TE, UK; 2 Department of Epidemiology, Johns Hopkins Bloomberg School of Public Health, Baltimore, MD 21205, USA; 3 School of Public Health, Imperial College, London, UK; 4 Guangzhou No. 12 Hospital, Guangzhou, Guangdong 510620, People's Republic of China; 5 School of Public Health, The University of Hong Kong, Hong Kong SAR, People's Republic of China; 6 Department of Microbiology, The University of Hong Kong, Hong Kong SAR, People's Republic of China; 7 International Institute of Infection and Immunity, Shantou University Medical College, Shantou, Guangdong 515031, People's Republic of China

**Keywords:** influenza, mathematical modelling, social mixing, contact diary, travel, infectious disease transmission

## Abstract

A dense population, global connectivity and frequent human–animal interaction give southern China an important role in the spread and emergence of infectious disease. However, patterns of person-to-person contact relevant to the spread of directly transmitted infections such as influenza remain poorly quantified in the region. We conducted a household-based survey of travel and contact patterns among urban and rural populations of Guangdong, China. We measured the character and distance from home of social encounters made by 1821 individuals. Most individuals reported 5–10 h of contact with around 10 individuals each day; however, both distributions have long tails. The distribution of distance from home at which contacts were made is similar: most were within a kilometre of the participant's home, while some occurred further than 500 km away. Compared with younger individuals, older individuals made fewer contacts which tended to be closer to home. There was strong assortativity in age-based contact rates. We found no difference between the total number or duration of contacts between urban and rural participants, but urban participants tended to make contacts closer to home. These results can improve mathematical models of infectious disease emergence, spread and control in southern China and throughout the region.

## 1. Introduction

For many respiratory infections, spread is thought to occur predominantly through close person-to-person contact [1–5] There has been considerable interest in quantifying these interactions, particularly in understanding how different age groups mix and the extent to which mixing is assortative by age [6]. This interest has been driven by the role human contact patterns play in determining the effectiveness of vaccination and social distancing measures, and in the ability of mathematical models to predict the course of epidemics and the effectiveness of interventions [7]. However, empirical studies of social mixing specifically targeted at understanding the spread of respiratory infections have been restricted to European countries [6,8], the USA [9], Vietnam [10] and Taiwan [11]. These studies have measured the distribution of number of daily contacts, the proportion of contacts made within various social and environmental settings, as well as other properties of contacts thought to be important for direct transmission of infectious disease, such as duration and frequency of encounter, and whether the contact included touch or not. There is evidence that these self-reported contacts are relevant to the transmission patterns of acute respiratory infections, such as mumps, influenza, chickenpox and parvovirus [12–16]. While useful in its own right, studies thus far have not captured spatial aspects of social contact processes including spatial dispersal and heterogeneity, which may be useful in understanding the dynamics of contagion. Differences between urban and rural populations may be particularly important for the emergence and initial spread of zoonotic pathogens, as animal densities are generally higher in rural areas and urban locations tend to serve as hubs for global spread [17]. Spatial information on social contacts can increase our understanding of how acute respiratory infectious diseases spread regionally and provide an empirical foundation for models of the emergence, spread and control of novel pathogens of zoonotic origin, such as human-adapted avian influenza or severe acute respiratory syndrome (SARS).

China is home to over one-seventh of the world's population and is part of a region thought to play a critical role in global influenza dynamics [18]. Southern China has a high population density, is highly connected to regional and global population centres in terms of human and animal transportation and has been implicated in the emergence of SARS and H5N1 avian influenza [19,20]. During 2009 and 2010, we conducted a study of human contact patterns in a spatially random sample of communities in and around Guangzhou, China, a city of over 11 million and the capital of Guangdong province. We measured the quantity, duration, age group and distance from home of residence of contacts made by individuals aged 2 years and older. Here, we present the results of this study, contrasting contact patterns by age and community urbanization, and compare these results with those obtained for other countries.

## 2. Material and methods

### (a) Study population

Participants were recruited from randomly selected households, in 40 communities in a transect spanning a gradient of decreasing population density extending to the northeast of Guangzhou, China. Details on the methods can be found in Lessler *et al*. [21]. We define a community to be all of those within the jurisdiction of a single street or village committee (SVC), the smallest administrative unit in China. SVCs hold information on all residential households within their jurisdiction. A list of households was obtained from each SVC and then reordered randomly. Recruitment of households was attempted in sequence from this list until at least 20 households had been recruited into the study. The longitude and latitude of participating households were recorded by study researchers using a handheld GPS device.

Community locations were classified as urban or rural based on their administrative designation according the Chinese government. Communities were also classified by local population density. Local population density was considered to be the average of the density in the Landscan tile containing that location and all adjacent tiles (i.e. density in a 9 km^2^ area roughly centred on that community) [22]. Communities were divided into four categories based on the log of local population density: (i) low density—more than 1 s.d. below the mean, (ii) low-mid density—below the mean by less than 1 s.d., (iii) high-mid density—above the mean by less than 1 s.d., and (iv) high density—more than 1 s.d. above the mean.

### (b) Household and participant demographics

A consenting adult in each household was administered a household questionnaire on household composition, demographics, travel by household members, animal ownership and other household information. All consenting household members were administered a separate questionnaire collecting demographic information and recent travel history. Individuals were also asked to fill out a contact diary (see below). All questionnaires—household-level, individual-level and contact diaries—were administered as face-to-face interviews, with all responses recorded by study researchers. Parents were interviewed on behalf of children deemed too young to provide reliable information for both the individual participant questionnaire and the contact diary.

### (c) Contact surveys

Each consenting participant was asked to complete a contact survey. Contact surveys took the form of interviewer led questionnaires in which study participants report all the people they encountered the previous day (from waking to going to bed) with whom they had a face-to-face conversation or skin-on-skin touch; these types of contact are useful proxies for transmission opportunities [12–16]. We define a contact event as an event where contacts are reported, either as an encounter with an individual or a group, and count each individual contacted in such an event as a ‘contact’; hence, an participant's total number of contacts is the number of individuals reported across all contact events. However, we cannot determine whether all individuals are unique across contact events, and sometimes the same individual may appear in multiple events reported by a participant. Hence, the number of contacts reported should be considered a measure of the number of transmission opportunities that occurred through contact involving face-to-face interaction or touch, not necessarily the number of unique individuals who had the opportunity to infect (or be infected by) the study participant. To facilitate the reporting of high numbers of contacts, participants could report groups of similarly encountered individuals as a single event involving multiple people, rather than reporting each contact individually. This was to facilitate the recording of contact with large numbers of individuals and measure the right-hand tail of degree distributions with greater precision. Previous versions of contact diaries with single line entry for each contact (e.g. [6]) possibly suffer from the underreporting of contacts [8].There was no limit to the number of contacts (or contact events) that could be reported, and participants were not aware of how many slots for reporting were available on the questionnaire.

For each contact, participants were asked to report: the age range of the contact(s) (0–5 years, 6–19 years, 20–64 years, 65 years or older), whether the contact involved touch, the social context in which the encounter was made (the participant's home, work or school, travel, shopping, leisure or other), the total duration of the encounter (less than 10 min, 10–29 min, 30–59 min, 1 h or more) and the typical frequency of encountering the contact(s) (4 or more days a week, 2–3 days a week, once a week, less than once a week, met for the first time this day). For groups, participants were instructed to report the characteristics that would apply to the majority of group members.

Participants were asked to report the geographical location where each contact or group was encountered; individuals contacted could be recorded multiple times if they were encountered in multiple locations. Study staff performed an assessment of the study community and surrounding areas, recording key locations and landmarks in a KML file using Google Earth [23]. The study staff interviewed participants and used addresses, key locations and landmarks to determine the location (latitude and longitude) of each reported contact.

### (d) Estimating the duration of contact events

The total number of contacts is likely to be a crude approximation of those encounters’ potential to result in disease transmission. We use the total time in which individuals are exposed during contact events as an alternative metric and which may scale more closely with infection risk or exposure. Contact durations were reported as one of four categories; following Danon *et al.* [8], we assigned contact durations (an integer number of minutes; multiplying by the number of individuals if the event was with a group) following an exponential distribution to each contact event. We summed the duration of each reported contact event to find the total duration of all contacts for each participant. We estimated the distribution of contact time using an adaptation of the expectation–maximization algorithm to fit the exponential model. The actual duration of a contact event was considered to be within the reported range, or one of the two adjacent categories (for example, if an individual reported 10–29 min, the actual duration was considered to be between 0 and 59 min). Contacts were initially assigned a random duration within this interval based on an exponential distribution. This distribution was then re-estimated based on these times, and random contact times were reselected. This process was repeated until the distribution converged. In analyses, 100 parametric bootstrap iterations were performed in which contact times were randomly assigned in the same manner.

### (e) Statistical analysis

Age-based mixing matrices were calculated based on the ratio of the measured probability of a contact between individuals based on age group to a null model of the probability of that contact under an assumption of proportionate (random) mixing. Contact probabilities under the null model (i.e. proportionate mixing) were determined by the percentage of the population in each given age category in a 2009 national census of China, published in the China Statistical Yearbook 2010 [24]; also see the electronic supplementary material. Hence, values above one in the mixing matrix indicate more contact than expected between the two age groups, and values below one indicate less contact than expected. Confidence intervals were estimated by bootstrapping contact events (all contact events were sampled with replacement over 1000 iterations). Contact events where the respondent reported more than one age group for the contacts were dropped from analysis (1.2% of contact events).

Differences in contact profiles between groups were analysed using *χ*^2^-tests. Number of contacts, duration of contact and distance of contacts were converted into categorical variables as follows: numbers of contacts divided into categories with upper limits increasing by twofold (1–5, 6–10, 11–20, … , 320+), ages were divided into deciles (0–9, 10–19, … , 80+), total contact times into hours with upper limits increasing by twofold (0–1, 1–2, 2–4, … , 16+) and distances into approximate log categories (0–19 m, 20–124 m, 125–249 m, 250–499 m, 500–999 m, 1–2 km, 2–4 km, … , 326+ km). The resulting tables were then tested for significant non-independence using a *χ*^2^-test (simulated *p*-value, 10 000 iterations).

All statistical analyses were performed in the R statistical package (R v. 2.15, www.cran.org).

## 3. Results

We recruited 1821 participants from 856 households, across 40 communities. We achieved an overall household recruitment rate of 85.8% and an individual recruitment rate of 49.9%; recruitment was generally easier in rural locations (see the electronic supplementary material). Our study population are broadly representative though young children and 30–34-year-old adults are underrepresented, as are single person households. Overall, there were 12 147 unique contact events reported by participants, comprising 33 789 people encountered within unique contact events. Participants identified contact events, involving either face-to-face conversation or touch, which occurred in 4803 locations.

### (a) Number and duration of contacts

**Figure 1.**
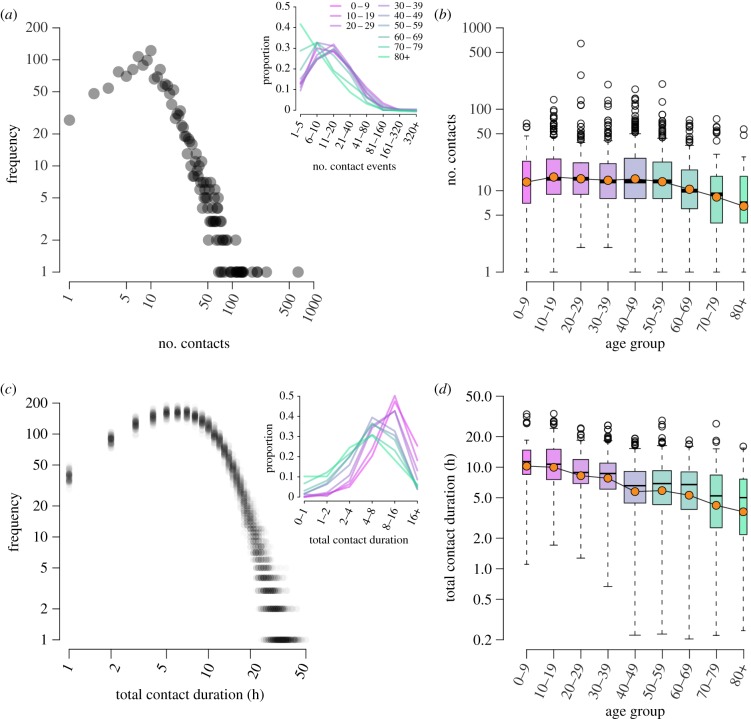
(*a*) The log–log distribution of number of contacts reported by participants. The inset shows the proportional distribution across log-binned contact number, split by age group of participant. (*b*) Boxplot of number of contacts reported by age group of participant; log-means are denoted by coloured circles. (*c*) The log–log distribution of total contact duration (rounded up to nearest hour); here, we show total durations from 100 re-samples with translucent points to illustrate the variation in assigned contact durations. The inset shows the proportional distribution across log-binned durations, by participant age group. (*d*) Total contact duration by age group. One participant reported zero contacts: they are in the 70-79 year age group and excluded from these plots.

We find the distribution of number of contacts made has a long right-hand tail, with several participants reporting more than 200 contact individuals (figure 1*a*). Only one participant reported zero contacts during their reporting day. While we find significant differences by age (figure 1), these differences are all owing to a significant decrease in contacts among older individuals (overall *χ*^2^ *p*-value less than 0.001, excluding those 70 and over, *p* = 0.14). There was no significant difference in the number of contacts when comparing administratively designated rural and urban study locations (*p* = 0.19). However, differences did exist when locations were stratified by population density (*p* < 0.001), with those living in high-density areas making significantly fewer contacts than those living in mid- or low-density areas (electronic supplementary material, figure S2). Individuals in the highest density areas tend to be significantly older (mean age 52 versus 44 overall), which in part explains this reduction, though in log-linear models a significant effect remains after adjusting for age, with those in the densest areas having 0.78 (95% confidence interval (CI): 0.68–0.90) times the number of contacts as those in other areas. See the electronic supplementary material, table S2, for further stratification of number of contacts by age group and contact characteristics.

When we consider the total time that participants spent with contacts, their total contact duration, we find a significant difference in duration between age groups and a steady decline in contact duration with increasing age (about 1.2%, per year figure 1*c*,*d*). Those aged 0–9 and 10–19 years have a similar distribution of contact durations. The decline in total contact duration begins as individuals enter their twenties. We find similar patterns in the data from a previous European-based contact survey, the POLYMOD study (electronic supplementary material, figure S4) [6]. However, the POLYMOD data showed a more truncated right-hand tail in number of contacts, but a longer right-hand tail for total contact duration, possibly owing to differences in study questionnaire design. We also find household contacts and those encountered most frequently dominate the daily contact duration for all age groups (electronic supplementary material, table S3).

### (b) Distance from home of social encounters

**Figure 2.**
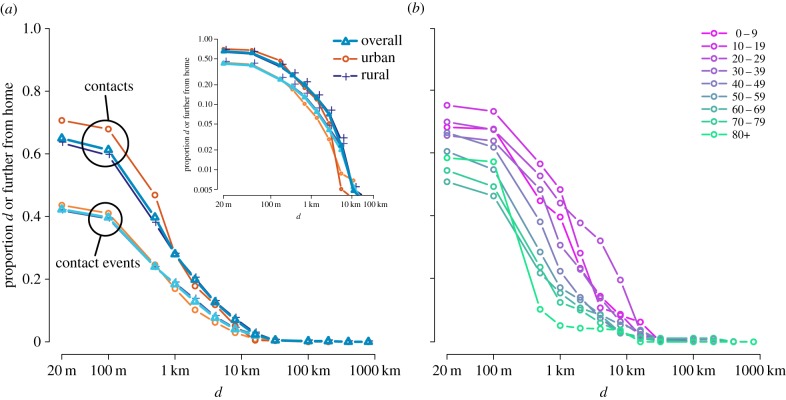
Inverse cumulative distance kernels for the number of contacts and number of contact events, showing the proportion of each made at (or further) particular distances from home. Distances are from the home location of each participant to the location where contact occurred, in kilometres. (*a*) Distance kernels for number of contacts and contact events, and those made by participants living in urban and rural locations (administratively defined); inset shows a linear-scale version. (*b*) Distance kernels for the number of contacts made for each participant age group.

The furthest distance at which a contact event occurred was 552 km from the participant's home location. The majority of contact events occurred in or near participants’ homes, with 45% occurring within the home and 58% occurring in the home or within 20 m (figure 2). However, these events accounted for only 35% of the person/contact pairs, i.e. home and near-home contact events were with smaller groups of people than contact events at larger distances. The contacts of older individuals were significantly more likely to occur in the home, and when they did occur outside the home tended to be closer than those of younger respondents. Residents of administratively designated rural areas were significantly more likely to have contacts in their home (37% versus 24%), but when they made contacts outside of their home, they tended to be further away compared with urban residents (upper quartile 2.7 km versus 2.0 km). When stratified by population density of home location, a slightly more nuanced picture emerges (electronic supplementary material, figure S3). The proportion of number of contacts in the household declines with increasing population density, from 38% in the lowest density locations to 17% in the highest density location. However, there was no clear trend in the distance from home of non-household contacts, with the contacts of participants residing in mid-density locations encountered the furthest distance from home (upper quartile low density: 1.9 km, low-mid: 2.6 km, high-mid: 2.6 km, high: 2.3 km).

### (c) Assortative mixing

**Figure 3.**
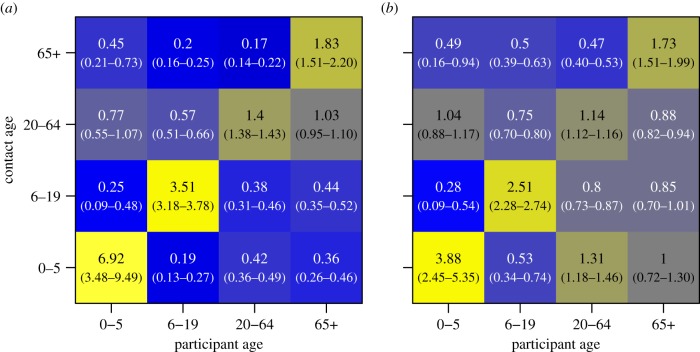
Age-based mixing matrices for (*a*) total number of contacts and (*b*) total contact duration. Values are the ratio of observed contact rates to those expected assuming proportionate mixing using national-scale demographic data. Bluer colours indicate less mixing between age groups than expected by random mixing, and yellower colours indicate more mixing. 95% CIs are shown in parentheses, derived from 1000 re-samples of participant contact diaries.

The ages of contacts were only measured in coarse age categories; despite this, assortativity by age was still evident. All age groups were significantly more likely to have a greater number of contacts with a member of their own age group than would be expected if mixing were at random (figure 3*a*). Younger (0–19 years old) and older (65+) participants were over three times as likely to have contact with individuals of their own age, while assortativity was weaker among 20–64 year olds, who were 1.4 times as likely to mix with those of their own age. When measured by contact duration, assortativity for each age group remains significant though slightly attenuated, the exception being the contact rate of young children encountered by adults aged 20–64, which increases to 1.3 times more than if mixing was random.

**Figure 4.**
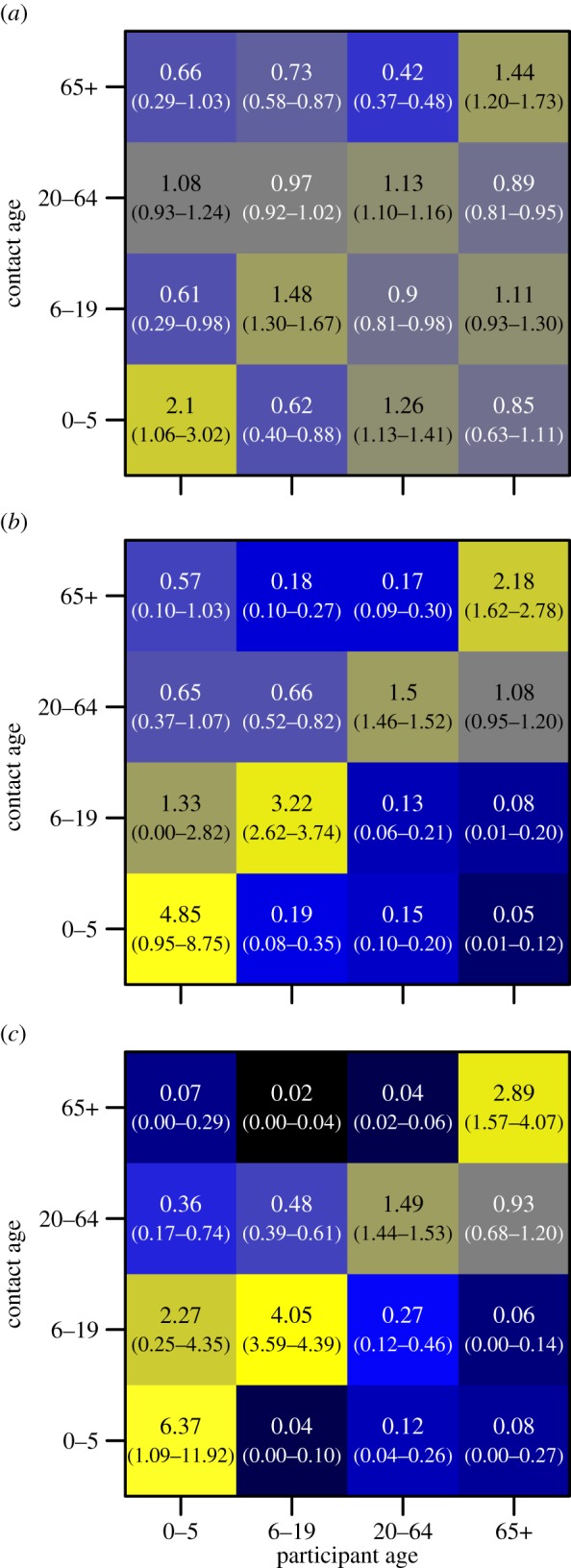
Age-based mixing matrices for total number of contacts, stratified by (*a*) within household, (*b*) non-household and less than 0.5 km from home, and (*c*) non-household and greater or equal to 0.5 km. The colour scales are the same as for figure 3.

When stratified by whether a contact was made within or outside of the household, we found assortativity by age to be stronger outside of the household (figure 4). Assortativity by age increases the further from home contacts are made. We found no qualitative difference in age-mixing patterns between urban and rural populations. Our measure of assortativity, relating number of frequency of contact reported to that expected by random mixing, may be biased if the demographic age structure of our study population differ from the larger scale demography on which our null models are based [25]; in light of this, we conducted a sensitivity analysis using complete age information for study households instead of national census data and found no significant differences (see the electronic supplementary material, figure S5).

## 4. Discussion

In a large, representative study of self-reported contact patterns in Guangdong province, China, we found patterns of contact broadly consistent with those observed in Europe and elsewhere in the world. However, some important differences were apparent. While European studies found that school-age children have the highest rates of contact [6], we found little difference by age group, except for a decreased number of contacts made by those older than 70 years. However, when we considered the total duration of contacts made, we found a steady decline with increasing age, a feature also present in European study data [6], though previously unreported (see the electronic supplementary material). From an epidemiological perspective, such contact patterns may be relevant to the transmission and control of influenza and other acute respiratory infections [4,12–16]. Although total contact number determines the potential frequency of exposure to infections, the risk of infection may depend more strongly on contact duration [4,8]. Modification of infection risk by time, not just frequency, of contact could be an important enhancement to models of infectious disease transmission.

Age-assortativity can have a fundamental impact on patterns of infection, and it has been suggested that these patterns could inform the targeting of control measures [16,26]. Age-assortativity in our population follows a similar pattern to that found in Europe and Vietnam [6,10]. We found age-assortativity to increase the further contacts were made from home. This may be explained by considering why individuals may travel different distances from home; our findings suggest contact with smaller, similarly aged groups of other individuals occur more often far from home. This is consistent with the finding that clustering of transitive links between contacts has been found to increase with distance from home in a UK-based study [8]. These results suggest the location of contact events and the age distribution of contacts are not independent and may need to be modelled jointly to appropriately capture transmission dynamics.

Older adults in our study had significantly different contact patterns than younger individuals. Not only do individuals over the age of 60 tend to make a lower number of contacts than the general population, but these contacts were of shorter total duration and occurred closer to home: European-based [6,8] and Asian-based studies [10,11] also reported that older age groups have lower contact rates than other age groups. The most mobile adult age group, those aged between 20 and 29 years, also had high rates of contact in terms of both number and duration: this age group may be expected to play an important role in the geographical spread of close-contact infections and may be an important age group to target for the containment and control of emerging infections.

Residents of rural and urban locations had similar numbers of contacts except in those locations with the highest population density, where the number of contacts was reduced. While this reduction is in part explained by a higher prevalence of older individuals in these locations, it may also be evidence of ‘urban isolation’ [27] in a rapidly developing region of China. There is also the possibility this reflects a sampling bias, as most urban contacts happen outside of the home, hence the most social individuals may be harder to capture in our study (because they are frequently absent from home). The most marked rural–urban differences are found in the locations in which contacts occur, with more contacts occurring within the home in rural areas, but contacts made outside of the home occurring further from the home. This is in contrast to a previous study of travel patterns in a different province of China [28] which found rural populations stayed significantly closer to home than more urban populations. The frequency of more distant contacts among those living in rural communities (2% are 25 km or further from home) suggest that an emerging pathogen arising in a rural population will have numerous opportunities to make long distance jumps, and that containment may be difficult.

Coarse measurements limit the precision of some of our results. The locations of contacts made far from home were measured with less spatial resolution than those made within the home or immediate neighbourhood (e.g. a contact made in a different province would be assigned to the centre of the town or city where that contact occurred). The age categories used here are coarse and differentially sized; hence, some measures of contact (e.g. the raw number in each group) might be biased towards assortativity. We address this concern by use of China national census data and calculation of relative contact frequencies compared to the expectation, given the size of the age categories. If the local population is not reflected by the population reported in the census, then this may bias our results; however, our study population is a good match to the census data, suggesting any such bias is likely to be small (see the electronic supplementary material). Allowing for the reporting of contacts in groups allowed us to avoid apparent truncation issues seen in other studies [6,8], but also made it difficult to determine how many unique individuals were encountered during the day and may have inflated the number of contacts reported. The duration of contact was also reported relatively coarsely, requiring a parametric approach to estimate total contact duration. There are some limitations regarding the day and dates of contact diaries collected. Participants were recruited and interviewed throughout the year, though relatively few interviews were conducted during February owing to holidays associated with Chinese New Year. Also, as a consequence of normal working practices, we collected fewer contact diaries about Thursdays, Fridays and Saturdays than the other days of the week.

Our study provides an important measure of social contact and travel patterns in a Chinese population specifically aimed at measuring factors related to infectious disease transmission. The results show that the patterns seen when only examining numbers of contacts may differ from those seen when considering the duration of contact, though broad trends remain similar. Researchers should carefully consider which factor, duration or number, is more important in disease transmission when incorporating contact patterns into their models. This study directly relates contact patterns of individuals to measures of urbanization (here population density and official designation). We find little difference in the number and duration of contacts, or the age-mixing patterns, between these populations, but we do find substantial differences in the spatial distribution of contacts. Our results suggest that measurable social contacts occur in similar numbers regardless of urbanization if locations share a cultural context; hence, observed differences in disease incidence between urban and rural locations [29,30] may be better explained by other factors: travel patterns, the structural properties of full contact networks or incidental contacts not easily measured or observed.

## Notes

All study protocols and procedures were approved by the Institutional Review Boards of the Johns Hopkins Bloomberg School of Public Health, Hong Kong University, the University of Liverpool and Guangzhou People's Hospital No. 12. All participants provided written informed consent (for children, this was provided by their parents).

## Data accessibility

Aggregate contact information at the individual-level is included in the electronic supplementary material. We request users register their use by contacting Derek Cummings (dcumming@jhsph.edu), so they may be informed of any updates. More detailed data, with individual contact event information, are available upon request.
